# Role of immediate early genes in the development of salivary gland organoids in polyisocyanopeptide hydrogels

**DOI:** 10.3389/fmolb.2023.1100541

**Published:** 2023-02-02

**Authors:** Paulien Schaafsma, Laura Kracht, Mirjam Baanstra, Anne L. Jellema-de Bruin, Robert P. Coppes

**Affiliations:** ^1^ Department of Biomedical Sciences of Cells and Systems, Section Molecular Cell Biology, University Medical Center Groningen, University of Groningen, Groningen, Netherlands; ^2^ Department of Radiation Oncology, University Medical Center Groningen, University of Groningen, Groningen, Netherlands

**Keywords:** xerostomia, salivary gland stem cells, matrigel, self-renewal, premature differentiation, PIC hydrogels

## Abstract

Human salivary gland organoids have opened tremendous possibilities for regenerative medicine in patients undergoing radiotherapy for the treatment of head and neck cancer. However, their clinical translation is greatly limited by the current use of Matrigel for organoid derivation and expansion. Here, we envisage that the use of a fully, synthetic hydrogel based on the oligo (-ethylene glycol) functionalized polymer polyisocyanopeptides (PICs) can provide an environment suitable for the generation and expansion of salivary gland organoids (SGOs) after optimization of PIC polymer properties. We demonstrate that PIC hydrogels decorated with the cell-binding peptide RGD allow SGO formation from salivary gland (SG)-derived stem cells. This self-renewal potential is preserved for only 4 passages. It was found that SGOs differentiated prematurely in PIC hydrogels affecting their self-renewal capacity. Similarly, SGOs show decreased expression of immediate early genes (IEGs) after culture in PIC hydrogels. Activation of multiple signalling pathways involved in IEG expression by β-adrenergic agonist isoproterenol, led to increased stem cell self-renewal capacity as measured by organoid forming efficiency (OFE). These results indicate that PIC hydrogels are promising 3D matrices for SGOs, with the option to be used clinically, after further optimization of the hydrogel and culture conditions.

## Introduction

The establishment of organoid cultures of multiple solid tissues derived from self-organizing adult stem cells has opened tremendous possibilities for regenerative medicine. Nevertheless, scarce human biopsy material often contains insufficient number of stem cells limiting their clinical potential (Coppes et al., 2009; Pringle et al., 2013; Nanduri et al., 2014). 3D organoid culture systems have been developed for many tissues, such as the brain (Clevers, 2016), liver (Broutier et al., 2017), pancreas (Boj et al., 2015; Georgakopoulos et al., 2020), and kidney (Schutgens et al., 2019). These 3D organoid culture systems allow the expansion of adult, tissue-specific stem cells that remain genetically and phenotypically stable over time (Li et al., 2020). However, most currently available protocols for organoid generation critically depend on the use of Matrigel, which is a not well-defined matrix and thus exhibits batch to batch variability. Moreover, its mouse-tumour-derived origin forms a roadblock to clinical transplantation of stem cells (Hughes et al., 2010).

During the past years, alternatives for Matrigel have been explored for organoid culture systems. Hydrogels that could be compatible for clinical translation can be derived from either naturally existing or synthesized material. Naturally derived materials can be obtained through the process of decellularization, used to rid the ECM of native cells and genetic materials while maintaining all relevant information necessary to instruct specific tissue formation and allow regeneration (Lin et al., 2014; Gilpin and Yang, 2017). Decellularized tissues have successfully been used as a scaffold to recreate various types of tissues (e.g., heart valve, esophagus, liver, lung) and are already used clinically (Dohmen, 2012; Parmaksiz et al., 2016; Gilpin and Yang, 2017; VeDepo et al., 2017). In addition, hydrogels derived from decellularized tissues enable endodermal organoid culture (Giobbe et al., 2019). Still hydrogels from decellularized tissues face several limitations similar to Matrigel such as batch-to-batch variability (Annabi et al., 2014). Broguiere et al. (2018) suggested that fibrin, another natural matrix derived from pooled plasma, which is already widely used in surgeries, could also be considered as an alternative to Matrigel (Li et al., 2015). This hydrogel of human-derived thrombin cross-linked fibrin gel supplemented with mouse laminin features mechanical as well as biochemical properties and supports long-term expansion of organoids derived from different mouse and human epithelial tissues. Although, mouse laminin shares 96% homology with human laminin it is still not of clinical grade and therefore this hydrogel is still incompatible with clinical use (Broguiere et al., 2018). Curvello et al. (2021) introduced the first engineered plant-based nanocellulose hydrogel for the growth of mouse small intestinal organoid. However, these plant-based nanocellulose hydrogels are not degradable in the human body simply because the lack of cellulases (Hu and Catchmark, 2011; Bačáková et al., 2014; Krüger et al., 2020). Synthetic hydrogels that are bioinert, biodegradable and allow expansion of stem cells under optimal, safe and good manufacturing practice (cGMP) are necessary alternatives for naturally derived matrices and subsequently enable translation of cell therapy to the clinic. In 2016, Gjorevski et al. (2016) reported the first synthetic hydrogel based on polyethylene glycol (PEG) which allowed expansion of mouse and human intestinal stem cells and organoid formation. Similar PEG-based hydrogels have demonstrated the ability to support the growth of liver organoids (Sorrentino et al., 2020). Although, in these hydrogels the presence of cell adhesion peptide RGD, a motif that is found in many ECM proteins and known to enhance cell binding and biocompatibility, is necessary (Gjorevski et al., 2016; Sorrentino et al., 2020). Nevertheless, it is a major disadvantage that the enzymatic digestion and mechanical disruption to recover cells from PEG-based hydrogels, is time-consuming and can damage cells and change their gene expression profiles (Zimoch et al., 2018).

Head and neck cancer (HNC) is the fifth most common cancer worldwide and accounts for more than 550,000 cases every year. Patients diagnosed with HNC will mainly be treated with radiotherapy, which significantly improves the survival rate of these patients (Lombaert et al., 2008; 2017; Nanduri et al., 2011; Srikantia et al., 2011). However, many critical organs and normal tissues that are sensitive to radiation (e.g., salivary glands (SGs)) are within the head and neck region and their exposure during radiation therapy of HNC still remains often unavoidable (Ülger et al., 2007; Pringle et al., 2016). As a result, SG dysfunction occurs and eventually result in hyposalivation-related xerostomia in 5.5%–46% of the patients (Villa et al., 2014). Xerostomia is associated with life-disrupting events such as impairment of taste, swallowing, and speech (Pradhan-Bhatt et al., 2013). Consequently, the quality of life is greatly impaired (Pringle et al., 2013; Nanduri et al., 2014). Current strategies to prevent or manage xerostomia are not satisfactory to treat radiation-induced SG damage (Nanduri et al., 2011; Ogundipe et al., 2021; Serrano Martinez et al., 2021; Zhao et al., 2021).

In 2008, we (Lombaert et al., 2008) showed for the first time that SG-derived stem cell transplantation restored saliva production after irradiation induced damage in mice. In addition, Pringle et al. (2016) showed a comparable effect after xeno-transplantation of human SG-derived stem cells. This preclinical model demonstrates that autologous adult tissue-derived stem cell therapy to repair radiation-induced normal tissue damage might be an alternative for (the lack of) long-term treatment (Lombaert et al., 2008; Pringle et al., 2016). Unfortunately, the clinical application of this treatment is also hampered by the reliance on Matrigel, hence the need of a GMP-compliant hydrogel for 3D organoid culture systems is emphasized once again.

In this study, we investigated the ability to culture and expand SG stem cell derived organoids using synthetic hydrogels based on the oligo (-ethylene glycol) functionalized polymer polyisocyanopeptides (PICs). PIC hydrogels mimic the architecture and mechanical properties of ECM proteins collagen and fibrin (Zhang et al., 2020). To improve biocompatibility and cell binding, the polymer can be further functionalized with well-known cell binding proteins, such as GRGDS (Op ’t veld et al., 2018; Zimoch et al., 2018; Zhang et al., 2020). Recent studies already showed that PIC gels allow mammary gland organoids (MGOs) formation from mouse mammary fragments or single mammary epithelial cells as well as to expand adult stem cell-derived liver organoids (for up to 14 passages) from two human donors (Ye et al., 2020; Zhang et al., 2020). However, the PIC polymer properties differed between the studies, suggesting that different matrix properties such as stiffness and cell binding site density affects the behaviour (e.g., spreading, migration, proliferation and differentiation) of each cell type differently (Hogrebe et al., 2018). Since the hydrogel is fully synthetic, it has a clearly defined and reproducible chemical composition and therefore organoid formation, and expansion of these adult stem cells could be developed into a cGMP compliant version. Moreover, it has already been showed that PIC hydrogels do not show any adverse effects *in vivo* (Op ’t veld et al., 2018). We therefore hypothesize that PIC hydrogels may be useful for SG-stem cell derived organoid expansion to overcome the limitations for clinical translation.

## Methods

Human biopsies of non-malignant submandibular salivary gland (SMG) tissue were obtained from donors, after informed consent and Institutional Review Board (IRB) approval, that underwent an elective head and neck dissection procedure at the University Medical Centre Groningen (UMCG) or Medical Centre Leeuwarden (MCL) after they were diagnosed with a squamous cell carcinoma of the oral cavity.

### PIC hydrogel synthesis and preparation

 PIC polymers were synthesized through a nickel (II)-catalyzed copolymerization of a tri-ethylene glycol functionalized isocyano-(D)-alanyl-(L)-alanine monomer and the azide-appended monomer 2. Variation of the catalyst to monomer ratio (1:1000 and 1:5000) allowed us to synthesize polymers with different polymer lengths, respectively 1K and 5K. The molar ratio used between monomer M1 and M2 was 99:1. To functionalize the polymer, RGD peptide was reacted with DBCO to obtain the complex DBCO-RGD, which was reacted to M2 monomers present on the PIC polymer backbone. Subsequently, the polymers decorated with cell-adhering peptides were purified. For gel formation, 3 ml optimal SMG medium, consisting of Dulbecco’s modified Eagle’s medium/F12 (DMEM/F12) [Gibco, 11320-074] containing 1% Pen/Strep antibiotics [Gibco, 15140-122], 1% glutamax [ThermoFisher Scientific, 35050038], 1x N2 [Gibco, 17502-048], 20 ng/ml EGF [Sigma, E9644], 20 ng/ml FGF2 [Peprotech, 100-18B], 10 μg/ml insulin [Sigma, I6634], 1 µM dexamethasone [Sigma, d4902], 10 µM Y-27632 [Abcam, ab120129], 50 ng/ml noggin [Peprotech, 120-10C], 1 µM A8301 [Tocris Bioscience, 2939], and 10% R-spondin1 conditioned medium, was added to the freeze-dried polymers to prepare stock solutions of 5 mg/ml. After soaking on a shaking platform at 4°C for 4 h, a clear solution was obtained. PIC with two different molecular weights, 1 kDa (PIC-1K) and 5 kDa (PIC-5K) and various PIC concentration (0.5, 0.75, 1, 2, and 3 mg/ml), resulting in a higher stiffness and lower porosity with increasing concentrations, were used.

### Human SMG cell isolation

**FIGURE 1:**
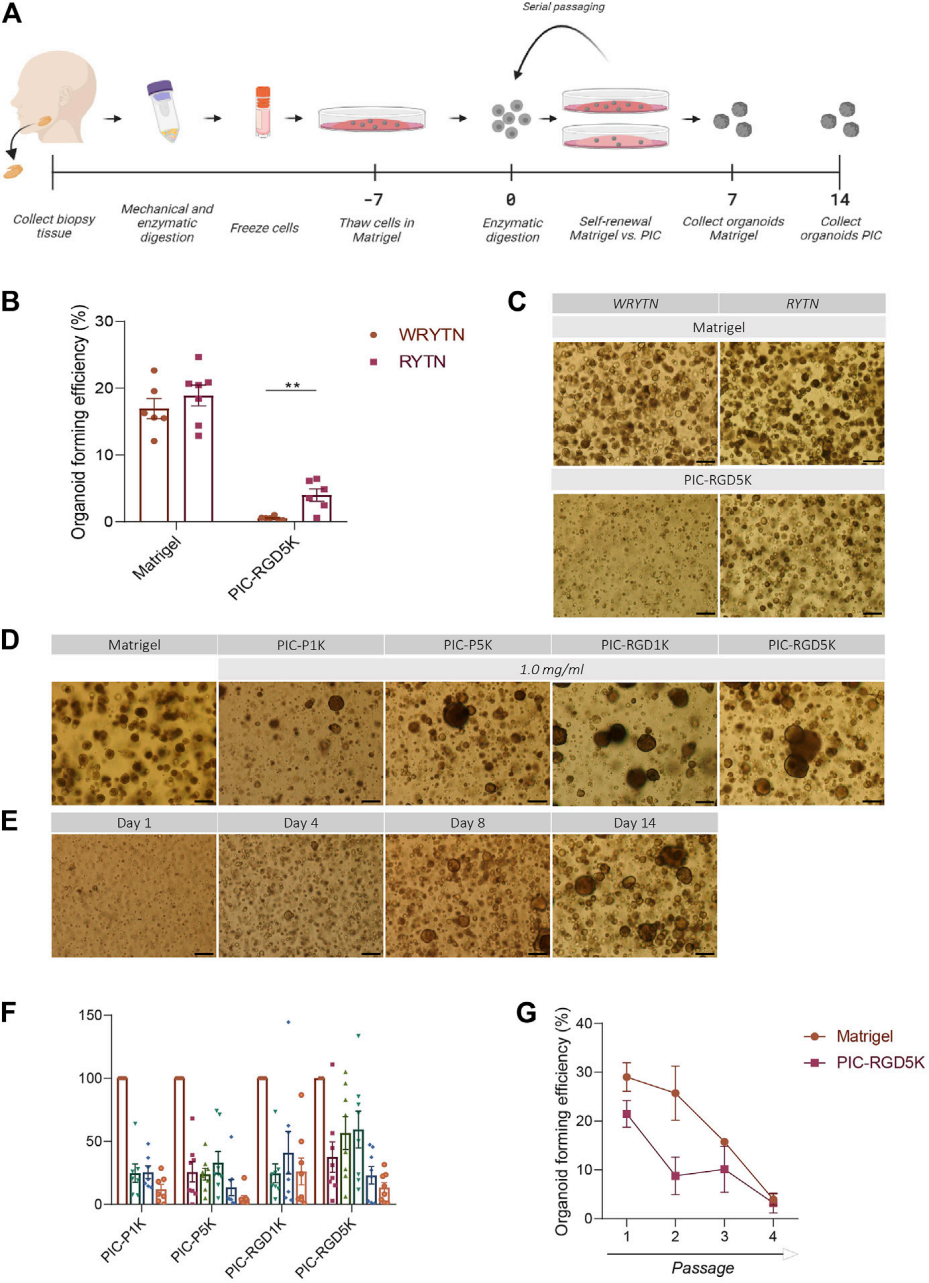
The organoid forming efficiency in PIC-cultured SGOs is reduced compared to Matrigel-cultured SGOs **(A)** Schematic overview of the study design **(B)** Organoid forming efficiency (OFE) of PIC-derived SGOs in WRYTN and RYTN media. Each symbol represents a different patient (*n* = 6). Data are represented as the mean ± SEM. Multiple t-test was used to test the differences between the groups (*p* = 0.0039). *p < 0.05, **p < 0.01, ***p < 0.001**(C)** Representative images of SGOs in the WRYTN and RYTN. Scale bar = 200 µM **(D)** Quantification of OFE in different polyisocyanopeptide (PIC) hydrogels with variated PIC concentrations (0.5, 0.75, 1, 2, and 3 mg/ml). Bars represent the mean of at least seven independent cultures ±SEM **(E)** Representative images of SGOs in Matrigel (7 days) and the different PIC hydrogels (14 days). Scale bar = 200 µM **(F)** Representative images of SGO formation in PIC-RGD5K over time. Scale bar = 200 µM **(G)** Organoid forming efficiency of SG-derived stem cells at different passages. Data are represented as the mean ± SEM.

 Human biopsies of non-malignant SMG were minced into small pieces using a sterile disposable scalpel followed by two rounds of mechanically dissociation using the gentleMACS dissociator [Milteny, 130-095-937] and enzymatic digestion in 5 ml HBSS [Gibco, 14175129] with 1% HSA [Sanquin, 15522636] buffer containing 125 µl Collagenase NB6/4 [Nordmark, N0002779], 62.5 µl Pulmozyme [KFF/Roche, RVG 16734] and 625 µl CaCl_2_ [Pharmacy UMCG, G00115] (per 100 mg tissue). Isolated cells were collected by centrifugation, washed twice with HBSS/1% HSA buffer filtered through a 100 μm cell strainer [Greiner, 542000] and again collected by centrifugation. Pelleted cells were resuspended in Cryostor^®^ CS10 [StemCell Technologies, 07931] with 10 µM Y-27632 at a final concentration of 4–10 million cells per ml, cryopreserved using a Corning^®^ CoolCell [Corning, 432000] which provides freezing at the rate of −1°C/min and stored at −140°C (Figure 1A).

### Human submandibular salivary gland organoid expansion

Cryopreserved cells were removed from the freezer, quickly thawed until a small clump of ice was left, washed twice with PBS/1% BSA and resuspended in DMEM/F12 containing 1% Pen/Strep antibiotics and 1% glutamax. 25 µl of cell suspension containing 400,000 cells, was mixed with 50 µl Matrigel^®^ [VWR, 35623] and seeded as a 75 µl drop in the middle of a well of a 12-well plate. Thirty minutes after seeding, culture medium was added. Culture media used in this study are described as follow: WRYTN, DMEM/F12 containing 1% Pen/Strep antibiotics, 1% glutamax, 1x N2, 20 ng/ml EGF, 20 ng/ml FGF2, 10 μg/ml insulin, 1 µM dexamethasone, 10 µM Y-27632, 50 ng/ml noggin, 1 μM A8301, 10% R-spondin1 conditioned medium and 50% Wnt3a conditioned medium, RYTN, DMEM/F12 containing 1% Pen/Strep antibiotics, 1% glutamax, 1x N2, 20 ng/ml EGF, 20 ng/ml FGF2, 10 μg/ml insulin, 1 µM dexamethasone, 10 µM Y-27632, 50 ng/ml noggin, 1 μM A8301, and 10% R-spondin1 conditioned medium. Where indicated, isoproterenol was added to the medium at a concentration of 1 µM. One week after seeding, salivary gland organoids (SGOs) were released from Matrigel^®^ by adding 1 mg/ml dispase [Gibco, 17105-041] for 1 h at 37°C, processed to single cells using 0.05% trypsin-EDTA [Invitrogen, 25300-096] and re-seeded in a 50 µl Matrigel^®^ or PIC hydrogel drop (15,000 cells per gel) to start a new passage. SGOs (14 days) were removed from PIC hydrogels by simply adding cold PBS (Figure 1A). At the end of each passage organoid and cell number were noted and used to calculate Organoid Forming Efficiency (OFE%) and Population Doubling (PD) as follows:

$$OFE\%=\frac{Number\;of\;organoids\;harvested\;at\;the\;end\;of\;the\;passage}{Number\;of\;single\;cells\;seeded\;at\;the\;beginning\;of\;the\;passage}\times 100$$

$$PD=\frac{\ln\left(harvested\;cells/seeded\;cells\right)}{\ln2}$$

### Human SGOs differentiation

Organoids recovered from Matrigel^®^ and PIC were quantified by counting to prepare a suspension of 30–40 organoids in 25 µl medium and 50 µl Matrigel^®^, and seeded as 75 µl droplets in pre-coated wells (40 µl 1:1 diluted Matrigel^®^). After solidification, 150 µl differentiation medium (DM) was added. DM was based on DMEM/F12 containing 1% Pen/Strep antibiotics and 1% glutamax, 10% FCS and supplemented with 1x N2, 20 ng/ml EGF, 20 ng/ml FGF2, 10 μg/ml insulin, 1 µM dexamethasone, 100 ng/ml FGF10 [Peprotech, 100-26], 50 ng/ml HGF [Peprotech, 100-39], 1 µm DAPT [Sigma, D5942], 200 nM Carbochol [Sigma, C4382] and 100 ng/ml Heparin sodium salt 0.2% [StemCell Technologies, 7980]. Medium was refreshed every 3–4 days for 3 weeks.

### RNA isolation and qRT-PCR

RNA was isolated from SGOs cultured in Matrigel^®^ (7 days) or PIC (14 days) using RNeasy Mini Kit [Qiagen, 74104] according to manufacturer’s instructions including DNase I [Qiagen, 79254] treatment. cDNA was synthesized from 500 ng mRNA using M-MLV Reverse Transcriptase [ThermoFisher Scientific, 28025013]. Quantitative PCR was performed in triplicate with the indicated primers (listed in Table 1), Bio-Rad iQ SYBR Green Supermix [Bio-Rad, 170880], and a Bio-Rad CFX Connect Real-Time PCR Detection System. Absolute data were first normalized to YWHAZ and subsequently to the Matrigel^®^ samples.

**TABLE 1:** List of primers used in this study.

	Forward primer	Reverse primer
*AMY*	TCA​CCA​TTG​GGT​TCT​GCT​GG	GAG​AGA​CCT​GAA​CCC​CTC​CA
*AQP5*	GCT​CAC​TGG​GTT​TTC​TGG​GT	CTT​TGA​TGA​TGG​CCA​CAC​GC
*KRT5*	GAG​ATC​GCC​ACT​TAC​CGC​AA	TGC​TTG​TGA​CAA​CAG​AGA​TGT
*KRT19*	TGG​AGA​TGC​AGA​TCG​AAG​GC	CTC​AGC​GTA​CTG​ATT​TCC​TCC​T
*MIST1*	CAG​CGG​ATG​CAC​AAG​CTA​AA	TAG​TTC​TTG​GCC​AGC​GTG​AG
*MUC7*	ATC​GTC​ACT​GTG​CTC​ATC​AGG	CTG​ATG​TCT​CCT​GGT​GCA​GTG
*SOX2*	TGG​CGA​ACC​ATC​TCT​GTG​GT	GGA​AAG​TTG​GGA​TCG​AAC​AAA​AG
*YWHAZ*	GAT​CCC​CAA​TGC​TTC​ACA​AG	TGC​TTG​TTG​TGA​CTG​ATC​GAC

### Bulk RNA sequencing

Human SGOs cultured in PIC or Matrigel^®^ were collected 7 and 14 days after passaging (at passage 2). RNA was isolated from the collected organoids and also from non-malignant submandibular gland tissues derived from three different human donors using the RNeasy Mini Kit according to manufacturer’s instructions including DNase I treatment. The quality and concentration of isolated RNA was determined with the Agilent TapeStation system. All samples were of high quality with an RNA integrity value >7.90 ng RNA was used as input from each sample for the QuantSeq 3′ mRNA-Seq Library Prep Kit [Lexogen, 015.96]. Briefly, RNA was incubated with oligodT primers containing the Illumina-specific Read 2 linker sequence to reverse transcribe RNA into cDNA. After RNA removal, second strand synthesis was performed using UMI-tagged random primers. Magnetic beads were used for size selection and to purify the double-stranded libraries. The libraries were amplified to add the complete adapter sequences. Libraries were equimolarly pooled and 1.8 p.m. of the pool with 15% PhiX were loaded on a NextSeq 500 [Illumina] for a 75 bp paired-end sequencing run.

### RNA sequencing analysis

All bioinformatic analyses were performed with available packages in RStudio (v4.0.2) and plots were generated with ggplot2 (v3.3.2) (Wickham, 2016). For PCA analysis logCPM counts (Supplementary Table S1) were used with the prcomp function of the stats packages (v4.1.2). The heatmap was created with pheatmap (v1.0.12) (Kolde, 2019), and row clustering was performed with the complete clustering method. For the differential gene expression analysis, lowly expressed genes (total count <1 in less than 2 samples) were excluded. The bioconductor package edgeR (v3.32.0) (Robinson et al., 2010)was used for normalization by using trimmed mean of M-values (TMM) and identification of differential expressed genes (logFC </>4, FDR<0.001) by fitting a generalized linear model. Gene ontology (GO) analysis for differentially expressed genes was performed with gProfiler with default settings (Raudvere et al., 2019). STRING analysis was performed with default settings (string-db.org) (Jensen et al., 2009).

To identify the enrichment of previously identified gene modules (unpublished data) in PIC- and Matrigel^®^ cultured SGOs, CPM values (Supplementary Table S1) of the present dataset of genes belonging to one gene module (Supplementary Table S2) were averaged per experimental group and presented as column row scores in a heatmap created with gplots (v3.1.1.) (Warnes et al., 2020).

### Statistical analysis

All statistical analyses in this study were performed using GraphPad Prism8 software (GraphPad, La Jolla, CA, United States). The number of patients analysed (n), presented error bars (SEM), statistical analysis and *p* values are all stated in each figure legend.

## Results

### An optimized PIC hydrogel supports salivary gland organoid expansion

Our previously reported 3D organoid culture system suffers from several shortcomings in its translation to the clinic for example by our optimized WRYTN medium. Hence, we previously developed a medium with a well-defined composition after risk assessment analysis had revealed that Wnt3a conditioned media, RSPO1 conditioned media, and Noggin did not meet the requirements for therapeutic use (unpublished data). In this new composition, Wnt3a is omitted as it is not yet available as a GMP-grade component. RSPO1, on the other hand, was replaced with human R-spondin-1 protein resulting in the so called GMP-grade expansion medium RYT, which performs similar to the “research” medium RYTN (unpublished data). Indeed, organoids in Matrigel showed a similar morphology and had the same size after omission of Wnt3a in the medium. Moreover, OFE was not affected. Interestingly, in PIC-RGD5K gels, the omission of Wnt3a in the medium led to an enlargement of the organoids while maintaining a round morphology and significantly improved the OFE (*p =* 0.0039) (Figures 1B, C). Based on these results further experiments were performed with RYTN medium.

Furthermore, a requirement for potential cell therapy is a 3D organoid culture system that allows expansion of stem cells using a GMP-compliant hydrogel. We assessed SGOs formation from Matrigel-thawed single human SG cells seeded in various PIC gels with RYTN medium. After 14 days in culture, we observed marginal organoid formation, with little to no proliferation when single cells were seeded in plain PIC resulting in an OFE below 50%, compared to Matrigel (Figures 1D, F). To stimulate organoid formation, we tested PIC hydrogels that were functionalized with RGD peptides. The incorporation of RGD motifs was not sufficient to increase organoid formation in the PIC-1K hydrogel, but it resulted in an average increase of 18% for PIC-5K hydrogels (Figures 1D, F). Although, the relative OFE in PIC-RGD5K was still lower compared to Matrigel after 7 days incubation, PIC-RGD5K consisting of 1.0 mg/ml PIC was identified to be most optimal for human SGOs (Figures 1E, F).

Next, we assessed the potential of PIC hydrogels to support long-term expansion of SG stem cells. Again, isolated single SG cells were thawed in Matrigel, recovered and embedded in PIC hydrogels at the same condition (1.0 mg/ml PIC-RGD5K). After 14 days, SGOs were harvested by simply adding cold PBS to break the gels. Subsequently, SGOs were enzymatically dispersed into single cells and reseeded into PIC-RGD5K. Repeated passaging in PIC-RGD5K resulted in SGOs formation which could be repeated up to 4 passages (Figure 1G).

### Organoids in PIC cannot be differentiated into mature SG cell lineages

**FIGURE 2:**
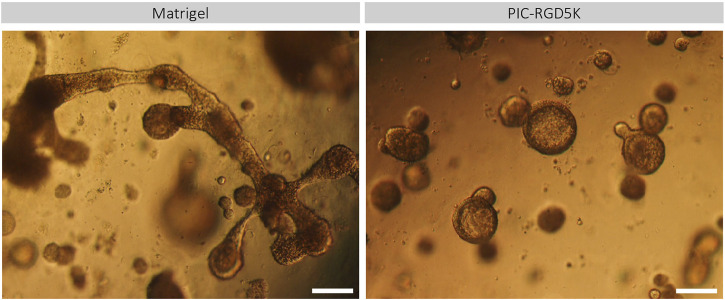
PIC-derived organoids cannot generate functionally mature SG cell lineages. Representative images of Matrigel- and PIC-derived SGOs at the end of the differentiation assay in Matrigel after 18 days in culture. Scale bar = 200 µM.

Another characteristic of human SGOs is that they should be able to form all different cell types of the tissue of origin. Previously it has been shown that human SGOs can be differentiated toward mature SG cell lineages when cultured in differentiation medium, void of inducers of proliferation. The potential of human SGOs cultured in PIC to generate functionally mature SG cell lineages was investigated. Light microscopy demonstrated that PIC-derived organoids preserved a more spherical shape and increased in size while Matrigel-derived organoids developed structures that contained both branching and lobular structures indicative of differentiation into SG structures (Figure 2). This data suggests that the differentiation potential in PIC-derived organoids is affected.

### Expression of immediate early genes is reduced in PIC-cultured SGOs

**FIGURE 3:**
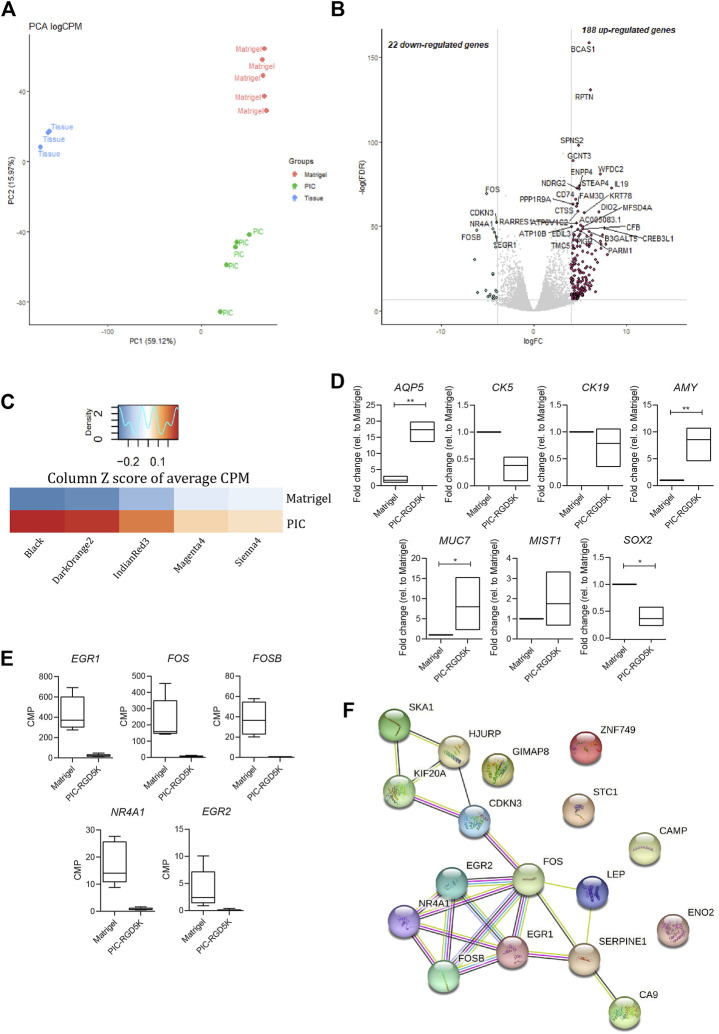
Polyisocyanopeptide (PIC)-cultured salivary gland organoids (SGOs) show reduced expression of immediate early genes and are in a more differentiated state than Matrigel-cultured SGOs **(A)** PCA plot of tissue, PIC- and Matrigel-cultured SGOs **(B)** Volcano plot depicting differentially expressed genes between PIC- and Matrigel-cultured organoids (−4>logFC>4, FDR<0.001). Red and green dots represent significantly up- and downregulated genes in PIC-cultured SGO, respectively **(C)** Average counts per million (CPM) of genes (Supplementary Table S1) belonging to gene modules (Supplementary Table S2) associated with stem cell-like (“Sienna4,” “Magenta3,” and “IndianRed3”) or differentiated (“Black” and “DarkOrange2”) trait identified in unpublished data in PIC- and Matrigel-cultured SGO depicted as column Z score **(D)** Expression of differentiation markers AQP5, AMY, MUC7, MIST1, stem cell marker SOX2 and ductal markers KRT5 and KRT19 in PIC- and Matrigel-cultured SGOs from three patients (*n* = 3). Multiple t-test was used to test the differences between the groups. *p < 0.05, **p < 0.01, ***p < 0.001 **(E)** Gene expression (CPM) of immediate early genes in Matrigel- and PIC-cultured SGO (*n* = 5) **(F)** STRING (Search Tool for the Retrieval of Interacting Genes/Proteins) analysis of the 22 downregulated genes in PIC- compared to Matrigel-cultured SGO.

To identify genes and associated pathways and functions that are differently regulated in human SGOs in PIC hydrogel and Matrigel, we performed bulk RNA-sequencing on fresh salivary gland tissue and SGOs cultured in Matrigel and PIC hydrogel. Principal component analysis (PCA) identifies a clear segregation of the tissue opposed to the organoids on PC1, identifying the difference of *in vivo* and *in vitro* conditions as the biggest driver of gene expression differences in this dataset. The second factor introducing variance in this dataset seems to be the difference in the culturing conditions, since PIC- and Matrigel-cultured organoids clearly separate on PC2 (Figure 3A).

1414 differentially expressed genes are identified between tissue and organoids (PIC and Matrigel together, Supplementary Table S3), confirming the difference between tissue and SGOs as seen in PC1 (Figure 3A). Clustering of these genes yields 3 clusters specific for tissue (cluster 2), PIC (cluster 1)- and Matrigel (cluster 3)-cultured SGOs (Supplementary Figure S1; Supplementary Table S4). Coherent with findings of the PCA analysis, clustering analysis indicates the expression of specific gene sets in PIC- and Matrigel-cultured SGOs.

When directly comparing the gene expression profiles of the two culturing conditions, 22 genes were depleted, and 188 genes were enriched in PIC-cultured SGOs compared to Matrigel-cultured SGOs (Figure 3B; Supplementary Table S5). To identify factors that are missing in PIC organoids compared to Matrigel organoids, we focused in the further analysis on the 22 genes found to be in PIC. Genes depleted in PIC-cultured SGOs were associated with GO-terms such as “response to oxygen-containing compound/lipid/hormone”, “skeletal muscle development”, “neurotrophic receptor tyrosine kinase 1 signalling,” whereas genes enriched in PIC were involved in amongst others “plasma membrane,” “extracellular space” and “immune response” (Supplementary Table S6).

To specify the biological processes in which the genes depleted in PIC-cultured organoids could be involved in, the present dataset was compared to a yet unpublished RNA-sequencing dataset of human salivary gland organoids. In the unpublished study, weighted gene co-expression analysis identified gene sets (modules) that are associated with different cellular states. Gene modules “Sienna4,” “Magenta3” and “IndianRed3” (Supplementary Table S2) positively correlated to a more primitive stem cell-like trait, whereas modules “Black” and “DarkOrange2” (Supplementary Table S2) positively correlated to a more differentiated trait. When compared to Matrigel-cultured SGOs, PIC-cultured SGOs were especially enriched in genes associated with gene modules correlated to the more differentiated cell state (Figure 3C). Indeed, expression of differentiation markers Aquaporin 5 (*AQP5*), α-Amylase (*AMY*), Mucin 7 (*MUC7*), and *MIST1* was increased while the stem cell marker *SOX2* and ductal markers *KRT5* and *KRT19* were decreased in PIC-cultured SGOs compared to Matrigel-cultured SGOs, as identified by qPCR (Figure 3D).

The genes that were found to be downregulated in PIC- compared to Matrigel-cultured SGOs mainly belong to the immediate early gene (IEG) family, such as *FOS*, *EGR*, and *NR4A1* (Figure 3E; Supplementary Table S1) and these genes also seem to form a highly interacting network (Figure 3F).

In contrast to our results described in “Organoids in PIC cannot be differentiated into mature SG cell lineages” and as depicted in Figure 2, our RNA-sequence data suggests premature differentiation of PIC-derived organoids, possibly due to the lack of IEG expression.

### Combined GSK3B and HDAC inhibition does not ensure for a more primitive stem cell state

To optimize a 3D organoid culture system that allows the production of a GMP-compliant cellular product, and due to the low OFE as a result of premature differentiation of SG stem cells, we aimed to further enrich the stem cell population in the organoid culture system. Therefore, we tested the addition of both the GSK3 inhibitor CHIR99021 (3 µM) and the histone deacetylase valproic acid (0.05 mM and 1 mM; VA) to the RYTN culture condition, a combination that previously improved the efficiency of organoid formation by stimulating Wnt and Notch signaling resulting in more Lgr5+ stem cells (Esfandiari et al., 2012; Yin et al., 2014; Pachenari et al., 2017). Unfortunately, we found that treating single cell seeded hSGSCs with CV did not enhance the OFE in both Matrigel and PIC (Supplementary Table S1).

### Stimulation of the IEG pathway by isoproterenol increased OFE in PIC-cultured SGOs

**FIGURE 4:**
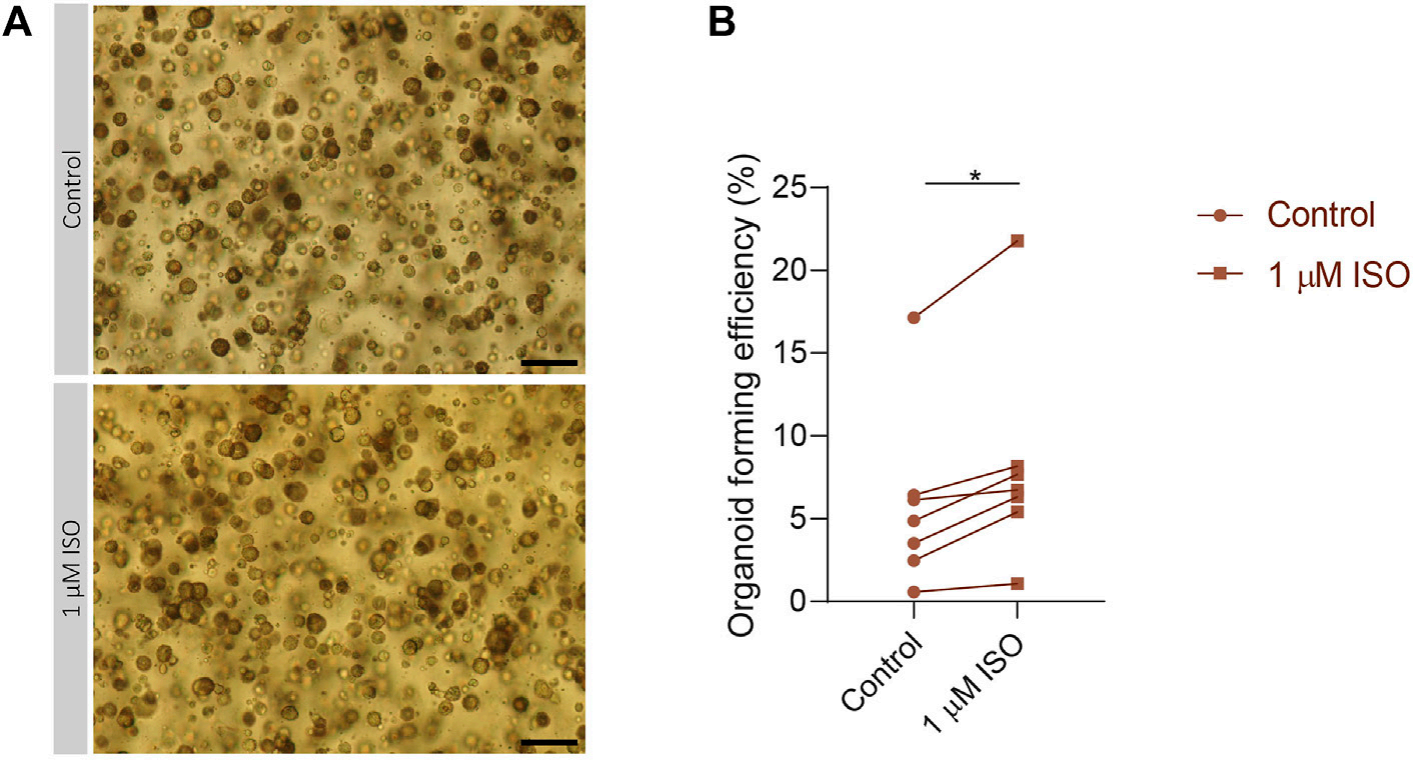
Isoproterenol increases OFE in PIC-cultured SGOs **(A)** Representative images of PIC-cultured organoids in culture treated with or without 1 µM isoproterenol. Scale bar = 200 µM **(B)** Organoid forming efficiency after treatment with 1 µM isoproterenol. Each dot represents a different patient (*n* = 7). Statistical significance (*p* = 0.0156) between the control and isoproterenol treated group was determined using Wilcoxon matched-pairs test. *p < 0.05, **p < 0.01, ***p < 0.001.

To validate the importance of the expression of IEGs for the capacity of SG stem cells to form SGOs, PIC-cultured SGOs were exposed *in vitro* to the β-adrenergic agonist isoproterenol, known to activate multiple signalling pathways involved in IEG expression (Yeh et al., 2012; Bahrami and Drabløs, 2016). Indeed, SGOs cultures exposed to isoproterenol demonstrated a significant increase in OFE compared with untreated cultures. A maximum increase of 5% was observed following 1 µM isoproterenol exposure, compared with untreated control cultures without isoproterenol (*p =* 0.0156*;* Figures 4A, B). Higher concentrations of isoproterenol (10 µM) did not show an effect on OFE while concentrations of 100 µM isoproterenol appeared to be toxic (data not shown).

## Discussion

Nowadays, Matrigel is the golden standard used in our and many other 3D organoid culture systems, however its poorly defined composition and animal origin hampers the utility of stem cells in clinical applications (Kim et al., 2022). PIC hydrogels have recently been implemented in multiple 3D organoid culture systems to allow the expansion of adult, tissue-specific stem cells *via* a potentially GMP-compliant procedure to make stem cell transplantation therapy clinically applicable. Here, we report that PIC hydrogels are sufficient to culture SGOs from encapsulated SG-derived stem cells. However, premature differentiation, probably as a result of decreased expression of IEGs, does not allow for unlimited expansion of stem cells. Modulation of pathways involved in the IEG expression using isoproterenol increased salivary gland stem cell potential.

We showed that within non-functionalized PIC hydrogels SG-derived stem cells display marginal organoid formation, with little to no proliferation. After incorporation of the RGD peptide, an increase in OFE was most abundantly observed in PIC5K hydrogels suggesting the importance of the presence of this motif in cell growth. PIC-RGD5K hydrogels with PIC concentrations between 0.5 and 1 mg/ml were most favorable for organoid proliferation. However, gels with PIC concentrations below 1 mg/ml softened within a few hours and no longer provided sufficient mechanical support, a crucial property for robust cell growth (Zimoch et al., 2018), highlighting the importance of having a stably crosslinked matrix for long-term organoid culture. Nevertheless, the optimized PIC-RGD5K gel only supported organoid cultures for up to 4 passages. Similar results were shown in other studies where cells only proliferated in PIC hydrogels after functionalization with the cell-adhesive RGD peptide or supplementation with lamininentactin (Ye et al., 2020; Zhang et al., 2020). Though, these PIC hydrogels did support long-term organoid cultures (≥10 passages) from mouse mammary gland fragments and cells as well as from liver stem cells isolated from biopsies of livers used for transplantation. Moreover, these PIC hydrogels efficiently supported expansion of mammary gland organoids while preserving their branching capacity when reseeded in Matrigel, and liver organoids which retained their differentiation potential as demonstrated by gene expression and functionality assays. Stem cell behavior is tightly regulated by a combination of both intrinsic and extrinsic cues. External cues are provided by the ECM (Khadilkar et al., 2020). The RGD peptide is one of the most commonly used molecules in synthetic matrices, that usually lack specific cell-recognition signals, to mediate cell adhesion of encapsulated cells to the ECM (Santos et al., 2014). This adhesive interaction of cells with ECM components initiates a wide range of intracellular signaling pathways which play a critical role in regulating self-renewal, pluripotency, differentiation and cell reprogramming of stem cells (Massia and Hubbell, 1991; Hogrebe et al., 2018; Stukel and Kuntz Willits, 2018; Liu et al., 2022). Importantly, the cellular response can be influenced not only by the presence but also by the RGD density of the matrices (Ruoslahti and Pierschbacher, 1986; Jeschke et al., 2002; Saux et al., 2011; Stevenson et al., 2013; Santos et al., 2014). We think that the ability to give rise to organoids and the maintenance of SGOs could be dependent on RGD density and that the difference in long-term expansion is explained by a difference in RGD concentration. Namely, our PIC gel contains 3.05 × 10^−5^ M RGD, whereas concentrations of 6.30 × 10^−5^ M have been used in the study of Zhang et al. (Zhang et al., 2020). Future effort should be focused on finding the optimal RGD density for SG-derived stem cells to efficiently expanding and long-term culturing of SG-derived stem cells.

Furthermore, we showed that the PIC-derived SGOs could not be differentiated toward functionally mature SG cell lineages when cultured in differentiation medium compared to Matrigel-derived SGOs. Our RNA-sequence analysis provided clear evidence that PIC and Matrigel-cultured SGOs were different. PIC-cultured SGOs were enriched in genes correlated to a more differentiated trait, in contrast to Matrigel-cultured SGOs. Additionally, specific SG markers, defining acinar cells, were also upregulated in PIC-derived SGOs compared to Matrigel cultured SGOs while stem- and ductal markers were found to be downregulated as validated by qPCR.

While potential to differentiate into particular cell lineages is a characteristic of stem cells, likewise, the ability to self-renew without differentiation as coordinated by biochemical as well as biophysical factors such as the softness or stiffness of the microenvironment is another important trait (Lv et al., 2012; Smith et al., 2018). Extracellular cues modulate cell growth primarily through the activation of signaling pathways, leading to activation of the expression of cellular immediate early genes (IEGs) (Lee et al., 2010; Bahrami and Drabløs, 2016). A possible explanation for premature differentiation is that some of these signaling pathways are affected (Román-Trufero et al., 2009). Indeed, we found that multiple early immediate genes, associated with cell growth/proliferation and cell death/survival functions, were downregulated in PIC cultured organoids suggesting an affected mechanotransduction (Zhou et al., 2015). Upon stimulation of the signaling pathway involved in the expression of IEGs with isoproterenol (Bahrami and Drabløs, 2016), the observed increase in organoid forming efficiency of PIC-cultured organoids point towards the potential to adapt the culture medium specifically for PIC gels to optimize stem cell self-renewal.

Altogether, PIC hydrogels are promising for the clinical translation of organoids due to the possibility to GMP-grade production by replacing chemicals and reagents of the gel synthesis with GMP-grade products. Nonetheless, follow up studies should focus on the ideal RGD density and the composition of the medium to allow Matrigel equivalent expansion and differentiation of tissue-derived stem cells and to prevent premature differentiation.

## Data Availability

The datasets presented in this study can be found in online repositories. The data presented in the study are deposited in the https://www.ncbi.nlm.nih.gov/geo/, accession number GSE220084.

## Supplementary material

Supplementary Figure 1 Gene expression differences between fresh salivary gland tissue and salivary gland organoids. Heatmap depicting row Z score of CPM of differentially expressed genes (DEGs) between tissue and SGO (PIC and Matrigel together, Supplementary Table S3). These DEGs form 3 clusters (Supplementary Table S4). CPM = counts per million, DEGs = differentially expressed genes.

Supplementary Figure 2 CHIR99021 (C) and valproic acid (V) treatment does not enrich for stem cells and does not influence OFE in PIC hydrogels. Organoid forming efficiency in Matrigel and PIC after treatment with CV. Bars represent the means of 3 independent experiments ± SEM.
