# Supplementary figures and images for: Role of immediate early genes in the development of salivary gland organoids in polyisocyanopeptide hydrogels
Source: Front Mol Biosci. 2023 Feb 2;10:1100541. doi: 10.3389/fmolb.2023.1100541 (PMC9932530; doi:10.3389/fmolb.2023.1100541)

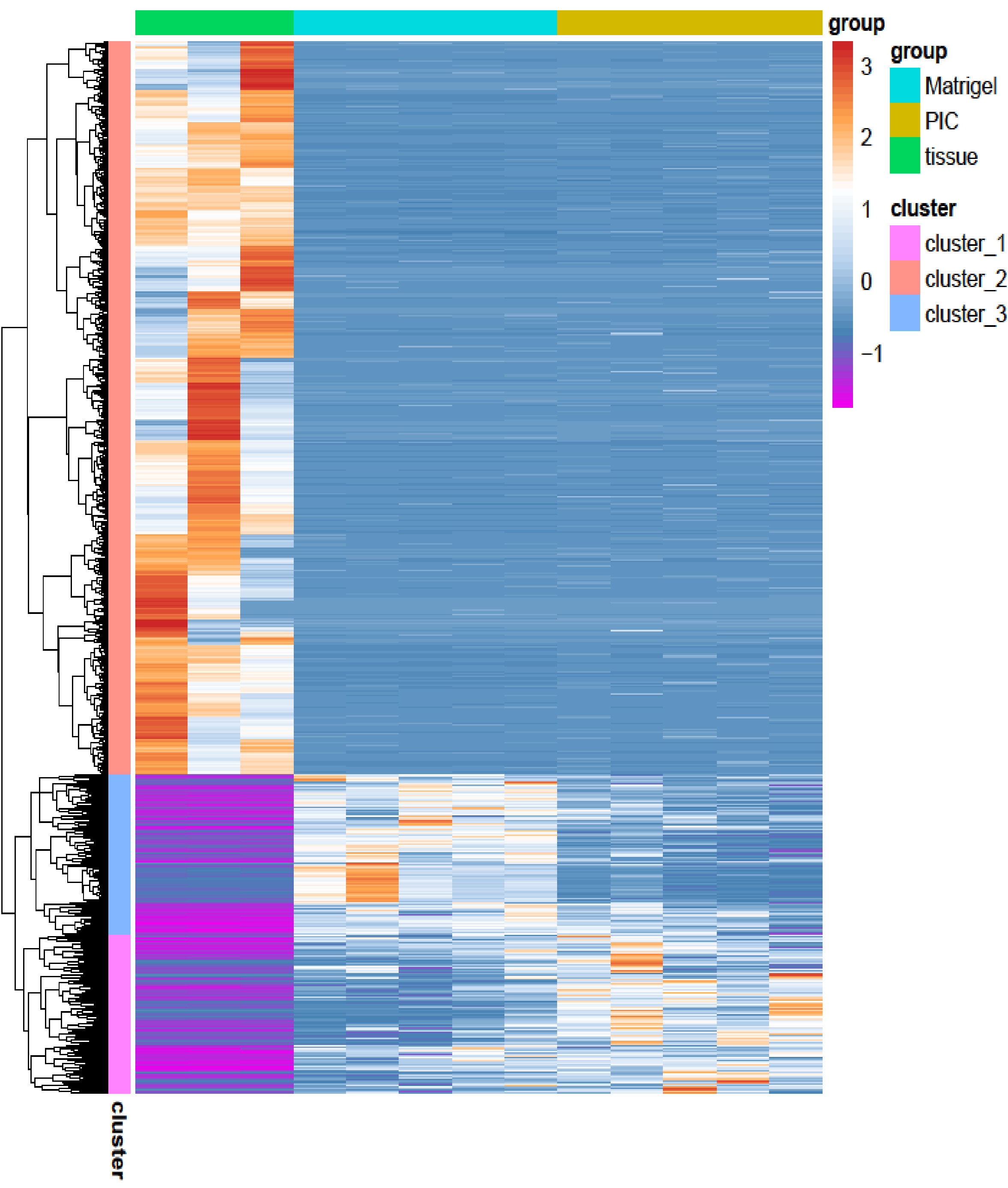

Supplement: Supplementary file 1 [file DataSheet1.zip › Supplementary Figure 1.JPEG]

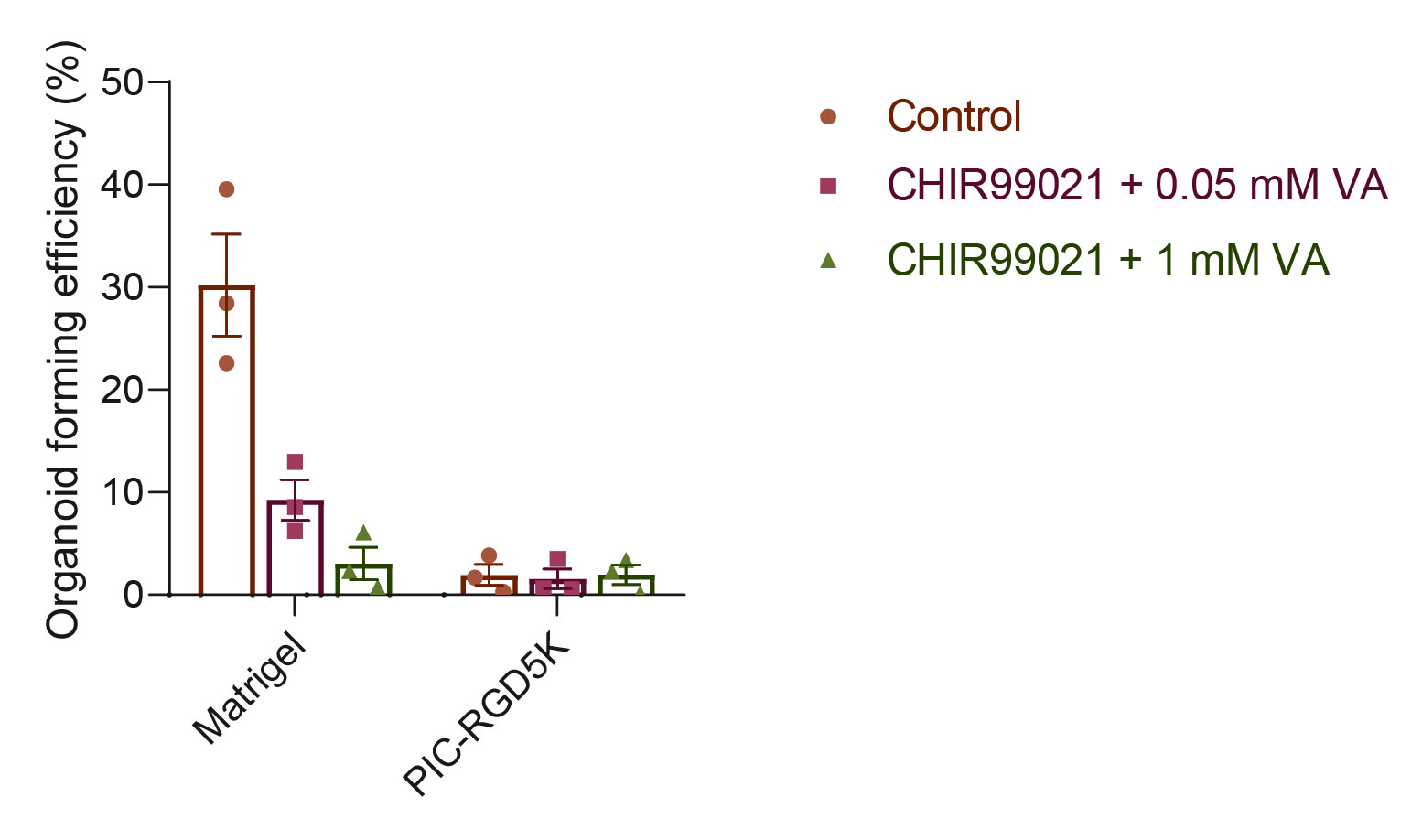

Supplement: Supplementary file 1 [file DataSheet1.zip › Supplementary Figure 2.JPEG]
